# Development of a Liquid Microbial Enzyme Synergistic Fermentation Process for Strong-Aroma and Soy-Sauce-Aroma Fresh Distiller’s Grains and the Evaluation of Their Nutritional Value in Pigs

**DOI:** 10.3390/ani16020170

**Published:** 2026-01-07

**Authors:** Caimei Wu, Ziyun Zhou, Meihong Li, Kun Zhang, Yixuan Zhou, Fali Wu, Jie Yu, Jian Li, Ruinan Zhang, Hua Li, Jiayong Tang, David Thomas, Lianqiang Che, Yang Lyu

**Affiliations:** 1 Key Laboratory for Animal Disease-Resistance Nutrition and Feedstuffs of China Ministry of Agriculture and Rural Affairs, Institute of Animal Nutrition, Sichuan Agricultural University, Chengdu 611130, China; 2 School of Agriculture and Environment, Massey University, Palmerston North 4410, New Zealand

**Keywords:** fresh distiller’s grains, microbial–enzyme synergy, liquid fermentation, growing pigs, SID, nutrient digestibility

## Abstract

China annually produces around 20 million tons of distiller’s grains, whose utilization is important for resource efficiency. These grains are rich in crude protein (CP), crude fiber (CF), and other nutrients, though their composition varies by grain type. This study applied a microbial–enzyme synergistic liquid fermentation process to two types—strong-aroma and soy-sauce-aroma grains—using a composite inoculant (*Lactobacillus*, *Saccharomyces cerevisiae*, *Bacillus*, each >1 × 10^9^ CFU/g) along with xylanase, cellulase, and protease. Optimal fermentation conditions were identified: for strong-aroma grains—water-to-grain material ratio 1.8, inoculum 0.25%, 25 °C, 16 h; for soy-sauce-aroma grains—ratio 1.8, inoculum 0.3%, 25.5 °C, 13 h. After fermentation, CP increased by 13.62% and CF decreased by 30.37% in strong-aroma grains, while in soy-sauce-aroma grains an 8.83% CP increase and 31.31% CF reduction were observed. Structural analysis of both grains showed looser fibrous formations and changes in protein secondary structure.

## Simple Summary

This study applied a composite microbial–enzymatic synergistic liquid fermentation process to strong-aroma and soy-sauce-aroma distiller’s grains. After fermentation, the crude protein content of both types increased significantly, while the crude fiber content decreased. Microstructural analysis showed improved structural characteristics. Fermentation also significantly enhanced the standardized ileal digestibility of most amino acids. The findings confirm that this process effectively improves the nutritional quality of distiller’s grains, supporting their utilization in pig feed.

## 1. Introduction

Distiller’s grains possess comprehensive nutritional value, being rich not only in crude protein (CP), crude fiber (CF), and ether extract (EE) but also containing essential amino acids such as lysine and methionine, along with relatively abundant vitamins and minerals. Variations in origin, raw material formulation, and batch of the grains lead to differences in nutritional content, a common drawback of most unconventional feed ingredients. The large amount of rice hulls added during the Baijiu brewing process results in high lignin–cellulose content [1], containing cellulose, hemicellulose, and lignin. Lignin is dispersed among cellulose fibers, while hemicellulose penetrates and connects lignin and cellulose fibers, forming a sturdy plant cell wall structure [2]. Hemicellulose, a non-cellulosic carbohydrate, decomposes into xylose, arabinose, glucose, galactose, and mannose, and is a main component of non-starch polysaccharides (NSPs). NSPs are typical anti-nutritional factors in grains, often affecting nutrient digestibility, increasing chyme viscosity, reducing digestive enzyme activity in the gut, and influencing gut microbiota [3]. Grzeskowiak et al. [4] demonstrated that dietary NSPs increase chyme viscosity, and Patience et al. [5] found that dietary NSPs reduce CP digestibility.

Liquid fermentation involves mixing feed with water at a ratio of 1:1.5 to 1:4.0 under controlled conditions, and adding microorganisms that produce enzymatically active metabolites during growth. These metabolites consume and degrade toxic substances or anti-nutritional factors in the feed, producing beneficial compounds like volatile fatty acids, vitamins, and bacteriocins. Studies have shown that liquid anaerobic fermentation of corn stalk silage produces feed rich in lactic acid and probiotics, significantly improving the resource utilization efficiency of corn stalks [6]. Missotten et al. found that feeding weaned piglets (27 days old) a liquid diet fermented with *Pediococcus acidilactici* increased average daily gain by 36.95% and significantly reduced the feed-to-gain ratio by 12.5% compared to a control group [7]. Fiona et al. used a compound microbial agent containing *Lactobacillus plantarum* and *Streptococcus lactisto* to ferment feed, and found higher lactic acid bacteria counts and lower *Enterobacteriaceae* counts in the liquid fermented feed [8]. Heres et al. used *Lactobacillus plantarum* to ferment feed for liquid feeding to broilers, and showed that high lactic acid bacteria content significantly suppressed *Salmonella* levels in the cecum [9]. Thus, liquid fermented feed can reduce feeding costs and improve animal performance. This study aimed to develop and optimize a liquid microbial–enzyme synergistic fermentation process for strong-aroma and soy-sauce-aroma fresh distiller’s grains and evaluate their nutritional value for growing pigs.

## 2. Materials and Methods

### 2.1. Materials and Instruments

The two types of fresh distiller’s grains (strong-aroma and soy-sauce-aroma) were obtained from Sichuan Province and Guizhou Province, China, respectively. The complex was purchased from Genyuan Bio-Company, Qingdao, Shandong, China. It contained *Lactobacillus*, *Saccharomyces cerevisiae*, and *Bacillus* (all >1× 10^9^ CFU/g), and composite enzymes including xylanase (enzyme activity > 500 U/g), cellulase (>500 U/g), and protease (>5000 U/g).

Key instruments included the following: Kjeldahl Nitrogen Analyzer (Hanon, Beijing, China), Automatic Fiber Analyzer (ANKOMA2000i, ANKOM Technology, Macedon, NY, USA), Oxygen Bomb Calorimeter (Parr6400, Parr Instrument Company, Moline, IL, USA), Automatic Amino Acid Analyzer (L-8900, Hitachi High Tech, Tokyo, Japan), High-Performance Liquid Chromatograph (e2695, Waters Corporation, Milford, MA, USA), Spectrophotometer (DU730, Beckman Coulter, Inc., Brea, CA, USA), Scanning Electron Microscope (Sigma360, ZEISS, Oberkochen, Germany), and Fourier Transform Infrared Spectrometer (Nicolet iS10, Thermo Fisher Scientific Inc., Waltham, MA, USA).

### 2.2. Optimization of Fermentation Process Parameters and Quality Assessment

#### 2.2.1. Single-Factor Optimization

A single-factor experiment with five factors at five levels was designed for each type of distiller’s grain, using total acid, total sugar, and hemicellulose content as response variables. Specifically, these factors are initial pH (5.2, 5.6, 6.0, 6.4, and 6.8), fermentation time (12, 16, 20, 24, and 28 h), feed-to-water ratio (1:1.8, 1:2.0, 1:2.2, 1:2.4, and 1:2.6), amount of bacterial enzyme added (1, 2, 3, 4, and 5%), and fermentation temperature (25, 30, 35, 40, and 45 °C). Each treatment had three replicates. The specific factor levels are shown in Table 1.

#### 2.2.2. Response Surface Methodology (RSM) Optimization

Based on single-factor results, a Box–Behnken central composite design was used with hemicellulose content (%) as the response value. The fitted quadratic polynomial equation is as follows: Y = a_0_ + a_1_A + a_2_B + a_3_C + a_4_D + a_12_AB + a_13_AC + a_14_AD + a_23_BC + a_24_BD + a_34_CD + a_11_A^2^ + a_22_B^2^ + a_33_C^2^ + a_44_D^2^.

Y is the dependent variable; a_0_ is the constant coefficient; a_1_, a_2_, a_3_, and a_4_ are linear coefficients; a_12_, etc., are interaction coefficients; and a_11_, etc., are quadratic coefficients. Design-Expert 13 software was used for data analysis and prediction. The predicted optimal values were experimentally verified.

### 2.3. Evaluation of Fermentation Effects

#### 2.3.1. Indicator Measurement

The nutrient composition was analyzed according to the following assay methods. Moisture, CP, EE, CF, ash, calcium (Ca), and phosphorus (P) were determined using the corresponding Chinese National Standards: GB/T 6435-2014, GB/T 6432-2018, GB/T 6433-2006, GB/T 6434-2006, GB/T 6438-2007, GB/T 6436-2018, and GB/T 6437-2018, respectively [10,11,12,13,14,15,16]. The neutral detergent fiber (NDF) and acid detergent fiber (ADF) contents were measured following GB/T 20806-2006 and NY/T 1459-2007, respectively [17,18]. Gross energy (GE) was determined using an automatic oxygen bomb calorimeter. Amino acid analysis included 17 hydrolyzed amino acids quantified according to GB/T 18246-2019, with tryptophan analyzed separately as per GB/T 15400-2018 [19,20].

#### 2.3.2. Ultrastructure Analysis

Dried samples were fixed on conductive tape, sprinkled evenly, and observed under scanning electron microscope after removing loose particles.

#### 2.3.3. Protein Structure Analysis

Samples were dried at 65 °C, ground, sieved (60 mesh), mixed with KBr (2 mg sample: 200 mg KBr), pressed into pellets, and analyzed by Fourier Transform Infrared Spectroscopy (FTIR) (resolution 4 cm^−1^, range 4000–400 cm^−1^, 32 scans). Spectra were deconvoluted using OMNIC v8.2 and plotted with Origin v10.1.

#### 2.3.4. Microbial Community Analysis

Samples (pre- and post-fermentation, triplicates) were stored at −80 °C and sent to Majorbio Co., Ltd. (Shanghai, China) for 16S rRNA sequencing. Alpha diversity (Chao, Ace, Shannon, Simpson indices), beta diversity (Principal Coordinates Analysis, PCoA), microbial composition, and functional prediction (Kyoto Encyclopedia of Genes and Genomes, KEGG) were analyzed on the Majorbio cloud platform (https://analysis.majorbio.com, accessed on 15 January 2024).

### 2.4. Determination of Standardized Ileal Digestibility of Amino Acids in Growing Pigs

#### 2.4.1. Experimental Design and Diets

Ten crossbred Duroc × (Landrace × Large White) barrows with an initial body weight of 22.46 ± 1.13 kg were fitted with T-cannulas at the distal ileum. Pigs were randomly assigned to a 5 × 5 Latin square design, including 1 nitrogen-free diet (NFD) and 4 test diets (unfermented and fermented distiller’s grains). The experiment consisted of 5 periods, each with 3 d adaptation, 3 d pre-feeding, and 2 d collection. Pigs were housed individually in metabolic cages (2.5 m × 1.8 m × 0.8 m), fed restrictively at 4% of BW, divided into three equal meals at 08:00, 14:00, and 18:00, and allowed free access to water. The room temperature was maintained at 26 °C. The nitrogen-free diet, strong-aroma and soy-sauce-aroma grain diet formulations are shown in Table 2.

#### 2.4.2. Sample Collection

Ileal digesta were collected continuously for 2 h post-feeding during collection days, frozen at −20 °C, freeze-dried, ground (40 mesh), and stored at −20 °C until further analysis. Diet samples were similarly processed.

#### 2.4.3. Calculations

Apparent ileal digestibility (AID) of amino acids (AA) was calculated as follows:

AID (%) = [1 − (AA in digesta/AA in diet) × (Cr_2_O_3_ in diet/Cr_2_O_3_ in digesta)] × 100

Basal ileal endogenous amino acid (BIAA) losses were determined from pigs fed the nitrogen-free diet.

Standardized ileal digestibility (SID) was calculated as follows:

SID (%) = AID + (BIAA/AA in diet) × 100

### 2.5. Determination of Nutrient Digestibility, Digestible Energy, and Metabolizable Energy in Growing Pigs

#### 2.5.1. Experimental Design and Management

Ten barrows (15.34 ± 0.55 kg) were randomly assigned to a 5 × 5 Latin square design with 5 diets (1 basal diet and 4 test diets containing the distiller’s grains). Each period had 3 d adaptation, 3 d pre-feeding, and 2 d collection. Pigs were housed individually, fed restrictively (4% BW, fed twice daily at 8:00 and 16:00), and managed similarly to previously described.

Diets included a corn-based basal diet (CSD, 68.52% corn) and test diets (WLD and FWLD) where distiller’s grains partially replaced portions of the corn. Vitamin and mineral premixes met NRC (2012) [21] requirements for 11–25 kg pigs. The diet formulation for soy-sauce-aroma grains is shown in Table 3.

#### 2.5.2. Sample Collection

Feces were collected for 2 days post-feeding, frozen at −20 °C, freeze-dried, ground, and stored at −20 °C until further analysis. Urine was collected into buckets containing 10% H_2_SO_4_, filtered, volumetrically measured, subsampled, and frozen at −20 °C.

#### 2.5.3. Calculations

Nutrient digestibility (%) = (Nutrient intake − Fecal nutrient output)/Nutrient intake

Diet DE (MJ/kg) = (GE intake − Fecal GE)/Feed intake

Diet ME (MJ/kg) = (GE intake − Fecal GE − Urinary GE)/Feed intake

Ingredient nutrient digestibility (%) = [100% × (Test diet digestibility − Basal diet digestibility)/Proportion of test ingredient nutrient in test diet] + Basal diet digestibility

Ingredient DE or ME (MJ/kg) = (Test diet DE/ME − Basal diet DE/ME × A)/B

A is the proportion of basal diet in test diet; B is the proportion of test ingredient; A + B = 100%.

### 2.6. Statistical Analysis

Data are presented as mean ± standard error (SEM). Data were organized using Excel 2019. The normality of residuals was confirmed using Shapiro–Wilk tests (all *p* > 0.05), and the homogeneity of variances was verified using Levene’s test (*p* > 0.05). Single-factor data were analyzed by one-way analysis of variance (ANOVA) using SPSS 24.0, with Duncan’s multiple comparison test (*p* < 0.05 significant). *t*-tests were used for two-group comparisons (*p* < 0.05 significant, 0.05 ≤ *p* < 0.10 a trend). RSM was used to analyze data using Design-Expert 12.0.0 for regression model construction and ANOVA.

## 3. Results

### 3.1. Single-Factor Optimization Results

**Figure 1:**
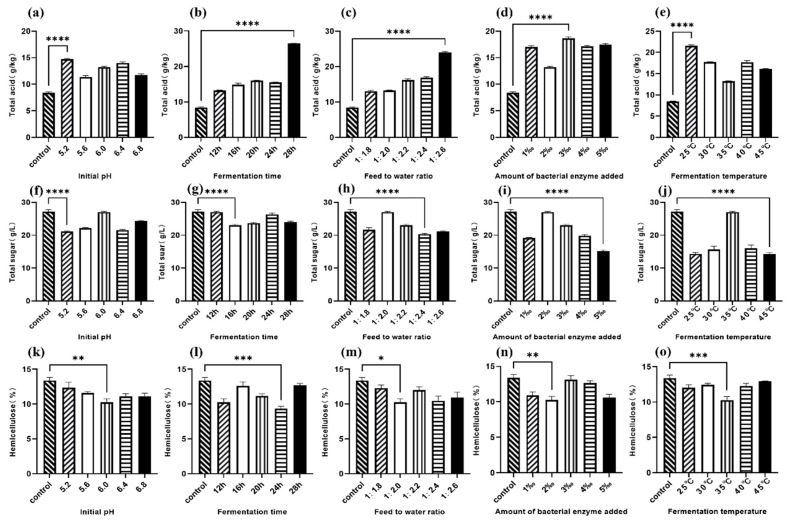
Single-factor experimental results after fermentation of strong-aroma grain. (**a**,**f**,**k**) Effect of initial pH; (**b**,**g**,**l**) Effect of fermentation time; (**c**,**h**,**m**) Effect of water-to-material ratio; (**d**,**i**,**n**) Effect of inoculant dosage; (**e**,**j**,**o**) Effect of fermentation temperature. * *p* < 0.10 (significant trend), ** *p* < 0.05, *** *p* < 0.01, **** *p* < 0.001.

**Figure 2:**
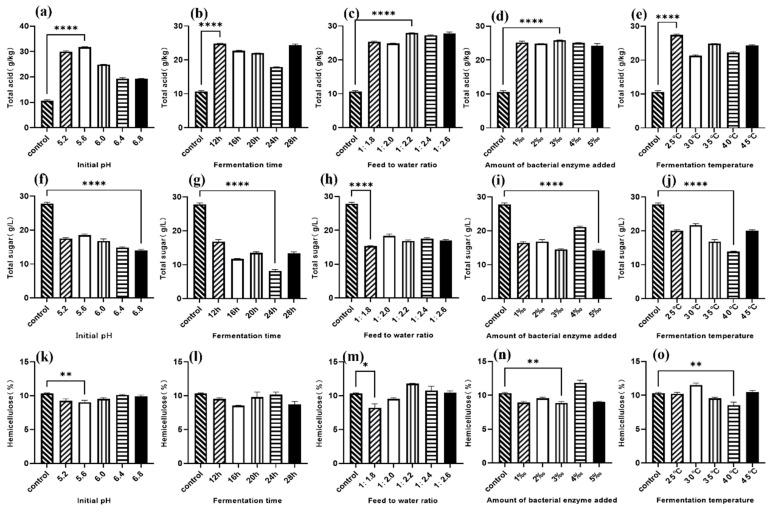
Single- factor experimental results after fermentation of soy-sauce-aroma grain. (**a**,**f**,**k**) Effect of initial pH; (**b**,**g**,**l**) Effect of fermentation time; (**c**,**h**,**m**) Effect of water-to-material ratio; (**d**,**i**,**n**) Effect of inoculant dosage; (**e**,**j**,**o**) Effect of fermentation temperature. * *p* < 0.10 (significant trend), ** *p* < 0.05, **** *p* < 0.001.

Figure 1 and Figure 2 show the single-factor results for strong-aroma and soy-sauce-aroma grains, respectively, based on total acid, total sugar, and hemicellulose content post-fermentation.

### 3.2. Response Surface Methodology Optimization Results

The quadratic polynomial equations for hemicellulose content (Y) after fermentation were derived. For strong-aroma grains, the equation is as follows: Y = 11.36 + 0.1708A + 0.2158B + 0.1367C + 0.3783D − 0.4225AB + 0.5675AC + 0.0875AD − 0.7975BC − 0.6975BD + 0.575CD + 0.1627A2 + 0.3302B2 + 0.2664C2 + 0.2589D2, factors A (fermentation time), B (water-to-material ratio), C (inoculant dosage), D (fermentation temperature).

For soy-sauce-aroma grains, the equation is as follows: Y = 9.88 + 0.1281A − 0.0271B + 0.0445C − 0.0272D + 0.35AB + 0.0276AC + 0.0236AD + 0.3695BC + 0.1884BD + 0.0806CD + 0.3316A^2^ + 0.0962B^2^ + 0.0379C^2^ + 0.0915D^2^, factors A (water-to-material ratio), B (inoculant dosage), C (fermentation temperature), D (fermentation time).

**Figure 3:**
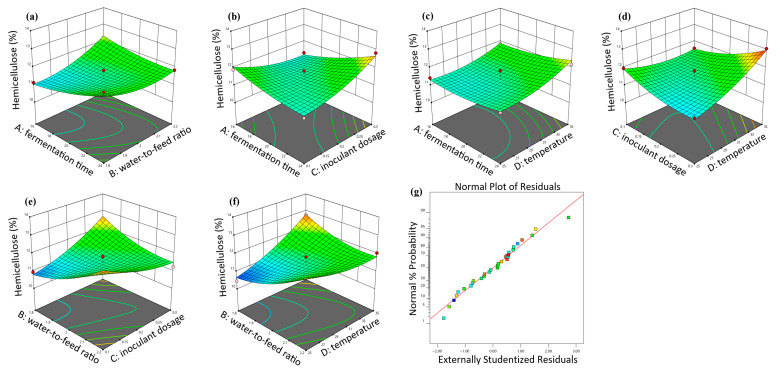
Response surface 3D plots showing the effect of fermentation conditions on the hemicellulose content of strong-aroma grain. (**a**) Interaction between fermentation time and water-to-material ratio; (**b**) Interaction between fermentation time and inoculant dosage; (**c**) Interaction between fermentation time and fermentation temperature; (**d**) Interaction between inoculant dosage and fermentation temperature; (**e**) Interaction between water-to-material ratio and inoculant dosage; (**f**) Interaction between water-to-material ratio and fermentation temperature; (**g**) Residual fit model plot.

**Figure 4:**
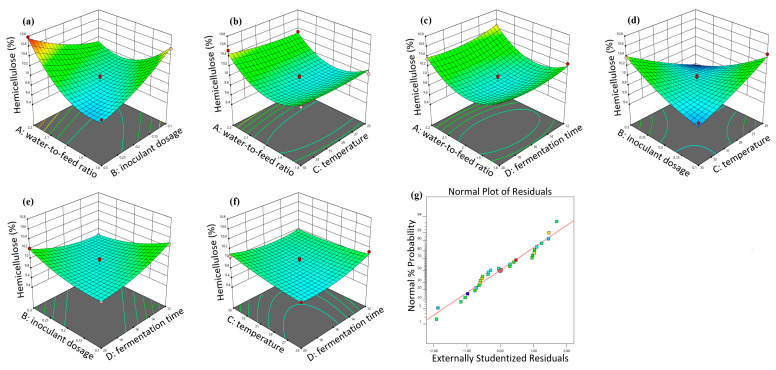
Response surface 3D plots showing the effect of fermentation conditions on the hemicellulose content of soy-sauce-aroma grain. (**a**) Interaction between water-to-material ratio and inoculant dosage; (**b**) Interaction between water-to-material ratio and fermentation temperature; (**c**) Interaction between water-to-material ratio and fermentation time; (**d**) Interaction between inoculant dosage and fermentation temperature; (**e**) Interaction between inoculant dosage and fermentation time; (**f**) Interaction between fermentation temperature and fermentation time; (**g**) Residual fit model plot.

The response surface plots for the interaction effects on hemicellulose content for strong-aroma grains are shown in Figure 3. The optimal conditions for strong-aroma grains were as follows: fermentation time 16.04 h, water-to-material ratio 1.82, inoculant dosage 0.26%, temperature 25.03 °C, minimizing predicted hemicellulose to 10.32%. The response surface plots for soy-sauce-aroma grains are shown in Figure 4. The optimal conditions for soy-sauce-aroma grains were as follows: water-to-material ratio 1.84, inoculant dosage 0.3%, temperature 25.58 °C, time 12.87 h, minimizing predicted hemicellulose to 8.21%.

### 3.3. Changes in Nutrient Content Before and After Fermentation

As shown in Table 4, microbial–enzyme synergistic liquid fermentation significantly affected both distiller’s grains. CP content increased by 13.62% strong-aroma grains, while CF decreased by 30.37% and 31.31%, respectively (*p* < 0.01).

Changes in amino acid content following fermentation are shown in Table 5. Tryptophan content tended to increase (*p* = 0.06) in strong-aroma grains and increased significantly (*p* < 0.01) in soy-sauce-aroma grains.

### 3.4. Effects of Fermentation on Ultrastructure, Protein Structure, and Microbial Community

**Figure 5:**
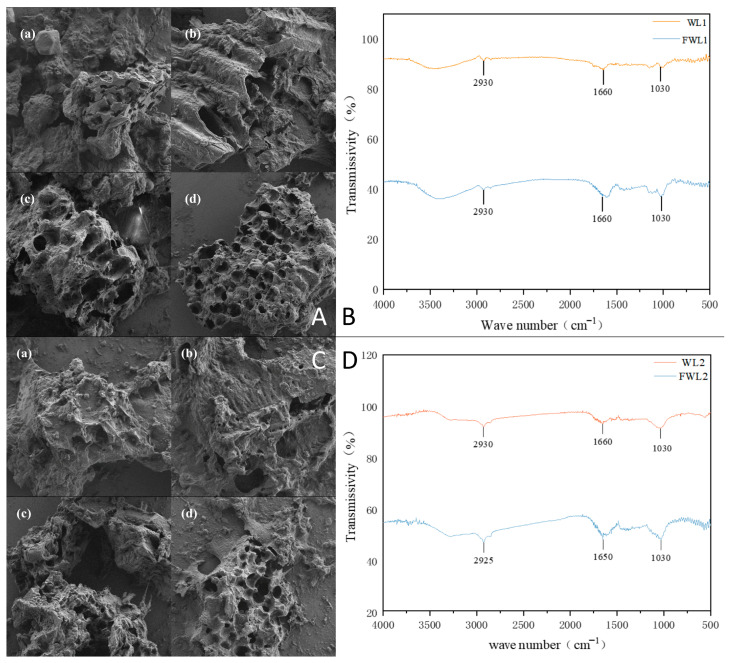
Comparison of ultrastructure and protein structure between two fresh distiller’s grains before and after fermentation. (**A**,**C**) SEM images (1000×; (**a**,**c**)): before fermentation; (**b**,**d**): after fermentation of strong-aroma (**A**) and soy-sauce-aroma (**C**) grains. (**B**,**D**): FTIR spectra of strong-aroma (**B**) and soy-sauce-aroma (**D**) grains.

Scanning electron microscopy (SEM) images (Figure 5A,C) showed compact structures pre-fermentation for both grains, which became loose and porous post-fermentation. FTIR spectra (Figure 5B,D) showed peaks mainly in the amide I band (1600–1700 cm^−1^). Post-fermentation, absorption intensity increased at ~1660 cm^−1^ (α-helix) and at 1060 cm^−1^ (C-O-C stretching of sugars). The peak at ~2930 cm^−1^ (C-H stretching) also intensified.

Alpha diversity indices are shown in Table 6. For fermented strong-aroma grains, Ace, Chao, and Sobs indices were higher, while Shannon and Simpson indices were lower compared to unfermented. For soy-sauce-aroma grains, Alpha diversity indices were similar pre- and post-fermentation. Coverage exceeded 99.9%, indicating reliable sequencing depth.

**Figure 6:**
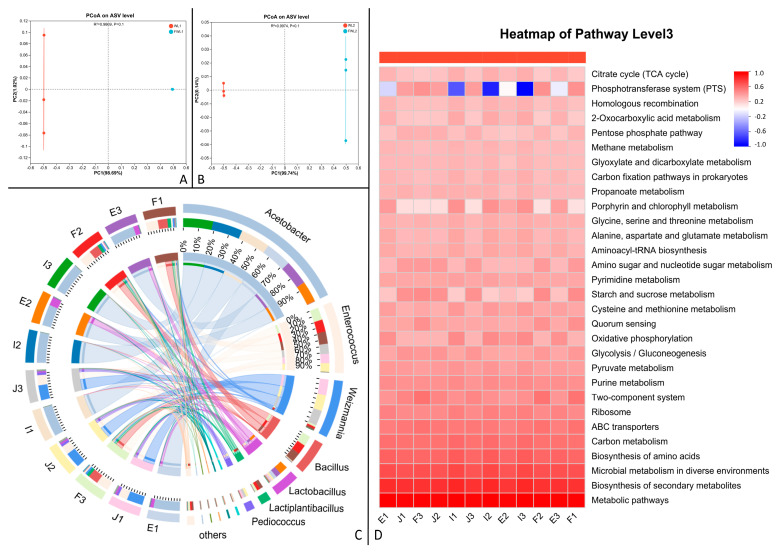
Comparison of microbial community between two fresh distiller’s grains before and after fermentation. (**A**) beta diversity PCoA plot of strong-aroma grain. (**B**) beta diversity PCoA plot of soy-sauce-aroma grain. (**C**) Circos plot of microbial composition. (**D**) heatmap of predicted microbial functions.

PCoA plots (Figure 6A,B) showed separation between microbial communities pre- and post-fermentation, though *p*-values were not significant. Circos plots (Figure 6C) revealed that the dominant bacterium pre-fermentation was *Acetobacter* for both grains. Post-fermentation, *Enterococcus* became dominant. *Bacillus* was relatively abundant in fermented strong-aroma grains, while *Weizmannia* was present in fermented soy-sauce-aroma grains. A KEGG functional prediction heatmap (Figure 6D) indicated positive correlations with pathways like two-component system, ABC transporters, carbon metabolism, amino acid biosynthesis, etc., post-fermentation. The phosphate transfer system correlation shifted from negative to positive after fermentation.

### 3.5. Ileal Amino Acid Digestibility in Growing Pigs

For strong-aroma grains (Table 7), fermentation significantly increased (*p* < 0.05) the AID of essential amino acids lysine, phenylalanine, and threonine. The SID of most essential (arginine, histidine, leucine, lysine, phenylalanine, threonine, valine, tryptophan) and non-essential (alanine, aspartic acid, glutamic acid, glycine, proline, serine) amino acids increased significantly (*p* < 0.05). For soy-sauce-aroma grains, AID significantly increased for all amino acids except arginine, lysine, and glycine (*p* < 0.05). SID significantly increased for all amino acids except lysine (*p* < 0.05).

### 3.6. Apparent Nutrient Digestibility, DE, and ME in Growing Pigs

As shown in Table 8, apparent nutrient digestibility of strong-aroma grains did not change significantly after fermentation. For soy-sauce-aroma grains, the apparent digestibility of all nutrients except acid detergent fiber increased significantly (*p* < 0.05) post-fermentation. DE and ME values increased significantly for both grains after fermentation.

## 4. Discussion

### 4.1. Fermentation Parameters and Post-Fermentation Quality Changes of the Two Fresh Distiller’s Grains

The results of this study demonstrate that liquid fermentation of fresh distiller’s grains using a composite microbial–enzyme preparation containing *Lactobacillus*, *Bacillus subtilis*, and *Saccharomyces cerevisiae*, combined with xylanase, cellulase, and protease, effectively degraded hemicellulose and increased CP content. Research has shown that *Bacillus subtilis* can degrade polysaccharides. Patel et al. reported that *Bacillus* consumes glucose and xylose, converting them to lactate [22], and xylose is a major component of hemicellulose. Another study also confirmed the polysaccharide-degrading role of *Bacillus*, where it was shown to produce cellulolytic and xylanolytic enzymes, effectively reducing the crude fiber content of rapeseed meal after fermentation [23]. The single-factor optimization results for the fermentation of fresh distiller’s grains in this study are consistent with these findings. The decrease in total sugar content is attributed not only to the action of cellulase and xylanase but also to the degradation of part of the polysaccharides by *Bacillus subtilis*, which saccharifies the enzymatically hydrolyzed small molecular carbohydrates to produce lactic acid, thereby increasing the total acid content in the fermented distiller’s grains. *Lactobacillus* is commonly used in feed ingredient fermentation and is itself an indispensable beneficial microbiota in the animal intestine. The most important role of *Lactobacillus* in the fermentation process is its ability to effectively control fermentation conditions. Its rapid reproduction leads to the production of large amounts of lactic acid in the fermentation environment, lowering the pH, inhibiting the growth of most contaminating bacteria, and improving the hygiene of the liquid fermentation process [24]. In a study by Anderson et al. on protein synthesis by yeast using glucose as a substrate, it was found that adding 25 mM inorganic phosphate increased protein synthesis by nearly 3.5 times [25]. Another study showed that fermentation of Huangjiu lees using *Candida utilis* and *Bacillus subtilis* increased CP by 14.5% [26]. In the current experiment, the CP content of the two types of distiller’s grains increased by 13.62% and 8.83%, respectively, consistent with the aforementioned research results. Variance analysis of the response surface optimization experiment for strong-aroma fresh distiller’s grains indicated the order of influence of the four factors on the response value as follows: fermentation temperature > water-to-material ratio > fermentation time > inoculum dosage. For soy-sauce-aroma fresh distiller’s grains, the order was as follows: water-to-material ratio > fermentation temperature > fermentation time > inoculum dosage. The greatest advantage of liquid fermentation compared to solid-state fermentation is the shorter fermentation time. Previous studies report that lactic acid bacteria in liquid fermentation show significant differences in lactic acid accumulation compared to the control group after just 8 h, and the lactic acid accumulation reached maximal levels and stabilized after 16 h [27]. The water-to-material ratio is a key condition distinguishing liquid from solid-state fermentation. During the fermentation of distiller’s grains, the overall trend in hemicellulose content change was an increase with a higher water-to-material ratio. This might be because *Bacillus subtilis*, which degrades hemicellulose, is an aerobic bacterium, and an excessively high water-to-material ratio reduces the oxygen content in the fermentation environment. Experiments fermenting corn stover with *Lactobacillus plantarum* and *Bacillus* showed that CF degradation rate at 95% moisture content was significantly lower than at 85% moisture content [28].

Observation via scanning electron microscopy revealed varying degrees of change in the surface structure of both types of distiller’s grains after fermentation. Following liquid fermentation treatment, the porosity increased, and the structure became looser compared to the original porous structure. Untreated distiller’s grains exhibited a compact and smooth surface, whereas the fermented grains showed a rougher surface and smaller particle size. This change might be related to the metabolites of the fermentation strains or the added protease [29], or it could be due to the degradation and disruption of fibrous materials by cellulase and xylanase during fermentation, altering the surface structure [30].

FTIR is a method combining Fourier transformation and infrared spectroscopy, commonly used to determine chemical functional groups, purity, and protein secondary structure, and is known for its high sensitivity, simplicity of operation, and detection sensitivity [31]. The infrared spectra of the distiller’s grains showed an enhanced absorption peak in the amide I band (1600–1700 cm^−1^) after fermentation. The amide I band is a marker band for protein secondary structure. For strong-aroma fresh distiller’s grains after treatment, the absorption peak shifted from around 1638 cm^−1^ to near 1600 cm^−1^. According to previously reported results, this suggests a change in the protein composition of strong-aroma grains, primarily characterized by a conversion involving arginine/glutamine. For soy-sauce-aroma grains, the absorption peak intensified at 1660 cm^−1^, which corresponds to the C=O stretching vibration of glutamine [32,33]. Both distiller’s grains showed a significant enhancement of the absorption peak at 2930 cm^−1^, attributed to C-H stretching vibrations. The peak intensification is likely due to the degradation of polysaccharides during fermentation, where chain breakage exposes more CH_2_ and CH_3_ groups [34]. The fermented soy-sauce-aroma grains also exhibited a more pronounced absorption peak near 1060 cm^−1^, influenced by the C-O-C ether bond stretching vibration of sugar units [35]. Combining the single-factor fermentation results, it is plausible that microbial metabolites or xylanase decomposed hemicellulose, breaking down polysaccharides into smaller oligosaccharides or altering their structure.

Microbial 16S rRNA high-throughput sequencing is used to study complex microbial communities, providing qualitative and quantitative (i.e., relative abundance) data through amplification and sequencing [36]. This study compared changes in the microbial composition of the feed substrate before and after fermentation through species abundance, alpha diversity, beta diversity, and KEGG functional prediction analysis. Species abundance plots showed that the predominant bacterium in the unfermented distiller’s grains was *Acetobacter*, while *Lactobacillus* and *Bacillus* were dominant in the fermented grains. Regarding species richness changes, the microbial abundance increased after fermentation for both distiller’s grains. This might be because the low pH of the untreated grains was unfavorable for microbial growth, whereas the post-fermentation environment was more suitable, allowing the inoculated *Lactobacillus* and *Bacillus* to proliferate. Combined with alpha diversity analysis and beta diversity PCoA plots, there was a clear trend for differences in microbial composition before and after fermentation for both distiller’s grains. The unfermented grains had a high ethanol content, and *Acetobacter* readily proliferates in ethanol-rich substrates, consuming ethanol and converting it to acetic acid. High concentrations of acetic acid can damage bacterial cell membranes, inhibit the synthesis of cellular metabolites, and affect bacterial reproduction [37,38,39]. This explains why the microbial diversity was relatively low in the untreated distiller’s grains before fermentation. After fermentation, the microbial community in the feed ingredients became more concentrated, with the strain composition stabilizing, primarily consisting of *Lactobacillus* and *Bacillus*. *Weissella* which was also present utilizes glucose as a carbon source to produce lactic acid and thrives at a pH of 5.5 or lower [40]. *Lactobacillus plantarum* can regulate sugar metabolism to produce more ATP and also alter amino acid metabolism in acidic environments [41,42,43]. *Weizmannia* belongs to the *Bacillus* genus, exhibits strong adaptability, and can survive in temperatures ranging from 35 to 50 °C [44]. The metabolites of *Weizmannia* can utilize polysaccharides and convert them to lactic acid [45].

According to the KEGG functional prediction abundance heatmap analysis of the distiller’s grains, the phosphotransferase system (PTS) showed a positive correlation in the microbial analysis of the fermented grains. Combined with the species abundance plot, the genes for the PTS likely originated from the Bacillus genus [46]. The PTS has very broad regulatory functions and is involved in nitrogen and carbon metabolism [47].

Based on the findings, a detailed hypothesis for the synergistic mechanism is proposed. Primarily, xylanase and cellulase initially degrade hemicellulose and cellulose, breaking the fibrous structure and releasing fermentable sugars. Subsequently, *B. subtilis* further decomposes these polysaccharides, while *S. cerevisiae* and *Lactobacillus* utilize the sugars for growth. *Lactobacillus* rapidly produces lactic acid, lowering the pH to inhibit pathogens and stabilize the system. This cooperative action collectively enhances fiber degradation, increases crude protein, and improves the overall nutritional quality of the fermented product. This synergistic interaction effectively disrupted the lignocellulosic matrix, increased crude protein availability, and modified the physicochemical structure of the substrate.

### 4.2. Ileal Amino Acid Digestibility, Nutrient Digestibility, Digestible Energy, and Metabolizable Energy of the Two Fresh Distiller’s Grains in Growing Pigs

The improvement in ileal amino acid digestibility was poorer for strong-aroma distiller’s grains compared to soy-sauce-aroma grains. This is likely due to the higher crude fiber and lower CP content of the strong-aroma grains. Arabinoxylan, β-glucan, and mannan in hemicellulose are major components of NSPs. Monogastric animals lack enzymes to digest NSPs, and NSPs increase chyme viscosity in the digestive tract, hindering contact between digestive enzymes and nutrients in the feed, leading to faster passage through the digestive tract and reduced digestibility and utilization of nutrients by the animal [48,49]. Upadhaya et al. compared the ileal amino acid digestibility of diets with and without added β-mannanase. Their results showed that the digestibility of lysine improved most significantly in the treated diet, and the digestibility of arginine, histidine, valine, and glycine also increased significantly [50]. Although only a few amino acids showed significant changes in AID for fermented strong-aroma grains in the current study, the improvement in SID for most amino acids was statistically significant. This might be due to the reduction in neutral detergent fiber content, which decreases endogenous amino acid losses in the ileum [51]. Similar to the AID and SID results, the horizontal comparison between the two grain types still showed less ideal apparent digestibility for the strong-aroma grains, most probably originating from their nutritional characteristics, high crude fiber, and low protein content. Related experimental studies have reported that *Lactobacillus* fermentation can alter the fiber structure of grains, thereby improving nutrient digestibility [52,53,54]. The raw material of soy-sauce-aroma distiller’s grains has a relatively high CP content. During fermentation, proteases and microbial metabolites influenced the protein structure, enhancing CP digestibility. Similarly, some studies have also found that *Bacillus subtilis* can alter the protein structure of soybean meal, increasing the acid-soluble protein content [55]. Shi et al. used *Bacillus subtilis* to ferment soybean meal, and their results showed increased CP digestibility of the fermented soybean meal [56], consistent with the findings of this study. The changes in DE and ME after fermentation were significant for both distiller’s grains. Studies have shown that DE and ME are negatively correlated with the content of neutral detergent fiber and acid detergent fiber, and positively correlated with CP content [57].

## 5. Conclusions

In conclusion, liquid microbial–enzyme synergistic fermentation improved the nutritional quality of both strong-aroma and soy-sauce-aroma fresh distiller’s grains. However, based on the comprehensive results of the feeding trial with growing pigs, soy-sauce-aroma fresh distiller’s grains show greater potential to replace traditional corn–soybean meal diets compared to strong-aroma fresh distiller’s grains, likely due to its lower crude fiber and higher CP content.

## Figures and Tables

**Table 1:** Single-factor design for the fermentation of each fresh distiller’s grain.

Factors	Level				
	1	2	3	4	5
Initial pH	5.2	5.6	6.0	6.4	6.8
Fermentation time (h)	12	16	20	24	28
Feed-to-water ratio	1:1.8	1:2.0	1:2.2	1:2.4	1:2.6
Amount of bacterial enzyme added (%)	1	2	3	4	5
Fermentation temperature (°C)	25	30	35	40	45

**Table 2:** Diet formulation and nutrient level (dry matter basis, %).

Ingredient	NFD	WLD1	FWLD1	WLD2	FWLD2
Corn starch	78.9	38.9	38.9	58.9	58.9
WL1	-	44.5	-	-	-
FWL1	-	-	44.5	-	-
WL2	-	-	-	24.5	-
FWL2	-	-	-	-	24.5
Sucrose	10	10	10	10	10
Soya-bean oil	3	3	3	3	3
Carboxymethyl cellulose	4	-	-	-	-
Calcium carbonate	0.5	0.5	0.5	0.5	0.5
Dicalcium phosphate	2.2	2.2	2.2	2.2	2.2
Cr_2_O_3_	0.3	0.3	0.3	0.3	0.3
NaCl	0.4	0.4	0.4	0.4	0.4
Vitamin premix ^1^	0.05	0.05	0.05	0.05	0.05
Mineral premix ^2^	0.15	0.15	0.15	0.15	0.15
K_2_CO_3_	0.4	-	-	-	-
MgO	0.1	-	-	-	-
Total	100	100	100	100	100
Nutrient level					
Crude Protein	0.28	3.59	3.65	5.93	6.11
Ca	0.53	1	0.76	0.8	1.16
P	0.45	0.48	0.44	0.42	0.41
Lysine	0.01	0.1	0.1	0.06	0.06
Methionine	0	0.02	0.02	0.02	0.02

NFD: nitrogen-free diet; (F)WL(D): (fermented) wine lees (diet); WL1: strong-aroma grain; WL2: soy-sauce-aroma grain. Note: ^1^ Vitamin premix (per kg of diet): VA, 15,000 IU; VD_3_, 5000 IU; VE, 40 IU; VK_3_, 5 mg; VB_1_, 5 mg; VB_2_, 12.5 mg; VB_6_, 6 mg; VB_12_, 0.06 mg; D-Biotin, 0.25 mg; D-Pantothenic Acid, 25 mg; Nicotinamide, 50 mg; Folic acid, 2.5 mg. ^2^ Mineral premix (per kg of diet): Fe (FeSO_4_·H_2_O), 100.0 mg; Cu (CuSO_4_·5H_2_O), 6.0 mg; Zn (ZnSO_4_·H_2_O), 80.0 mg; Mn (MnSO_4_·H_2_O), 3.0 mg; I (KI), 0.14 mg; Se (Na_2_SeO_3_), 0.25 mg.

**Table 3:** Dietary formulations and nutrient level of the soy-sauce-aroma fresh distiller’s grains before and after fermentation (dry matter basis, %).

Ingredient	CSD	WLD	FWLD
Corn	68.52	53.52	53.52
Soybean meal	27.88	27.88	27.88
WL	-	15.00	-
FWL	-	-	15.00
L-Lysine hydrochloride	0.45	0.45	0.45
DL-Methionine	0.10	0.10	0.10
L-Threonine	0.12	0.12	0.12
L-Tryptophan	0.05	0.05	0.05
Choline chloride	0.15	0.15	0.15
CaCO_3_	0.45	0.45	0.45
CaHPO_4_	1.15	1.15	1.15
Vitamin premix	0.03	0.03	0.03
NaCl	0.30	0.30	0.30
Cr_2_O_3_	0.30	0.30	0.30
Mineral premix	0.50	0.50	0.50
Total	100.00	100.00	100.00
Nutrient level			
Crude protein	17.93	20.73	21.21
Ca	0.63	0.74	0.82
P	0.42	0.51	0.54
Lysine	1.45	1.35	1.31
Methionine	0.15	0.16	0.17

CSD: corn-based basal diet; (F)WL(D): (fermented) wine lees (diet), which represents strong-aroma and soy-sauce-aroma grain (diet). Note: Premix compositions as in Table 3.

**Table 4:** Nutrient content of the two fresh distiller’s grains before and after fermentation.

Item %	UF	F	Change Rate	SEM	*p*	UF	F	Change Rate	SEM	*p*
**WL1**						**WL2**				
CP	10.19	11.57	13.62	0.03	<0.01	21.82	23.75	8.83	1.87	0.36
EE	12.27	15.96	30.07	0.39	<0.01	17.52	17.56	0.23	0.65	0.95
CF	22.83	15.89	−30.37	0.46	<0.01	15.74	10.81	−31.31	0.47	<0.01
NDF	44.05	31.02	−29.57	0.55	<0.01	40.02	32.16	−19.63	0.48	<0.01
ADF	30.81	20.2	−34.44	0.42	<0.01	29.95	22.06	−26.36	0.38	<0.01
Ash	8.27	10.32	24.79	0.27	<0.01	8.42	9.48	12.58	0.22	<0.01
Ca	0.1	0.15	55.12	0.02	0.07	2.2	1.82	−17.27	0.09	0.01
P	0.24	0.17	−30.13	0.01	<0.01	0.45	0.38	−15.67	0.06	0.31

UF: unfermented; F: fermented; WL1: wine lees 1, strong-aroma grain; WL2: wine lees 2, soy-sauce-aroma grain; CP: crude protein; EE: ether extract; CF: crude fiber; NDF: neutral detergent fiber; ADF: acid detergent fiber.

**Table 5:** Amino acid content of the two fresh distiller’s grains before and after fermentation.

Item %	UF	F	Change Rate	SEM	*p*	UF	F	Change Rate	SEM	*p*
**WL1**						**WL2**				
Arginine	0.34	0.36	5.88	0.07	0.86	0.64	0.69	7.81	0.07	0.54
Histidine	0.17	0.18	5.88	0.03	0.94	0.47	0.47	0.00	0.04	0.94
Isoleucine	0.30	0.31	3.33	0.05	0.87	0.73	0.91	24.66	0.08	0.17
Leucine	0.76	0.80	5.26	0.14	0.81	1.89	2.49	31.75	0.25	0.14
Lysine	0.19	0.19	0.00	0.01	0.84	0.47	0.39	−17.02	0.03	0.11
Methionine	0.14	0.14	0.00	0.03	0.98	0.36	0.29	−19.44	0.08	0.45
Phenylalanine	0.41	0.43	4.88	0.07	0.80	0.97	1.12	15.46	0.11	0.32
Threonine	0.28	0.29	3.57	0.05	0.85	0.86	0.83	−3.49	0.07	0.72
Tryptophan	0.14	0.17	21.43	0.01	0.06	0.14	0.21	50.00	0.01	<0.01
Valine	0.40	0.41	2.50	0.07	0.83	0.94	1.14	21.28	0.10	0.12
Alanine	0.57	0.58	1.75	0.09	0.92	1.41	1.78	26.24	0.17	0.09
Aspartic acid	0.57	0.59	3.51	0.10	0.84	1.43	1.57	9.79	0.13	0.34
Cysteine	-	-	-	-	-	0.15	0.17	13.33	0.04	0.59
Glutamic acid	2.04	2.12	3.92	0.34	0.82	3.52	3.98	13.07	0.39	0.30
Glycine	0.34	0.35	2.94	0.05	0.92	0.79	0.99	25.32	0.09	0.09
Proline	1.63	1.69	3.68	0.26	0.82	1.89	2.11	11.64	0.18	0.28
Serine	0.37	0.38	2.70	0.06	0.87	0.86	1.02	18.60	0.10	0.18
Tyrosine	0.37	0.38	2.70	0.07	0.90	0.86	0.93	8.14	0.09	0.45
Total amino acids	9.03	9.37	3.77	1.48	0.83	18.38	21.10	14.80	1.93	0.23

UF: unfermented; F: fermented; WL1: wine lees 1, strong-aroma grain; WL2: wine lees 2, soy-sauce-aroma grain.

**Table 6:** Alpha diversity analysis of microbiota in the two fresh distiller’s grains before and after fermentation.

Items	UF	F	SEM	*p*	UF	F	SEM	*p*
**WL1**					**WL2**			
Ace	25.99	179.70	16.94	<0.01	48.25	65.14	8.85	0.13
Chao	19.50	175.81	16.01	<0.01	46.33	64.39	8.78	0.11
Shannon	0.79	2.78	0.07	<0.01	0.48	2.36	0.03	<0.01
Simpson	0.61	0.13	0.05	0.01	0.84	0.17	0.01	<0.01
Coverage	1.00	1.00	0.00	0.05	1.00	1.00	0.00	0.08
Sobs	16.67	163.67	11.67	<0.01	38.33	63.00	6.86	0.02

UF: unfermented; F: fermented; WL1: wine lees 1, strong-aroma grain; WL2: wine lees 2, soy-sauce-aroma grain.

**Table 7:** Apparent and standardized ileal amino acid digestibility of the two fresh distiller’s grains before and after fermentation in growing pigs.

Items	AID				SID				AID				SID			
	UF	F	SEM	*p*	UF	F	SEM	*p*	UF	F	SEM	*p*	UF	F	SEM	*p*
	**WL1**								**WL2**							
CP	37.40	47.01	6.13	0.16	62.17	71.38	6.13	0.18	33.55	43.96	7.94	0.22	48.55	58.52	7.94	0.24
Arg	24.63	41.92	11.23	0.16	50.23	78.42	11.23	0.03	10.62	14.9	8.35	0.62	39.71	60.79	8.35	0.03
His	52.56	64.86	5.93	0.07	64.94	79.29	5.93	0.04	28.09	48.88	5.15	<0.01	40.54	64.11	5.15	<0.01
Ile	37.85	54.15	8.41	0.08	50.16	67.90	8.41	0.06	37.69	56.17	3.91	<0.01	45.91	65.96	3.91	<0.01
Leu	53.12	66.38	6.12	0.06	61.84	76.33	6.12	0.04	53.91	67.88	2.4	<0.01	59.24	74.10	2.40	<0.01
Lys	9.54	32.60	9.24	0.03	27.52	51.79	9.24	0.03	8.07	17.13	4.89	0.45	38.32	47.73	4.89	0.08
Met	35.19	53.90	11.28	0.13	43.62	61.56	11.28	0.14	32.31	48.83	3.55	<0.01	39.24	55.21	3.55	<0.01
Phe	48.66	65.07	6.91	0.04	58.12	74.75	6.91	0.04	43.93	64.42	3.6	<0.01	50.45	71.35	3.60	<0.01
Thr	24.06	45.18	7.80	0.02	45.47	73.11	7.80	0.01	18.61	40.01	5.53	<0.01	34.76	62.88	5.53	<0.01
Trp	48.87	62.40	7.46	0.10	62.89	82.08	7.46	0.03	19.7	50.23	9.8	0.01	39.92	69.12	9.80	0.01
Val	48.06	63.78	5.71	0.02	59.82	77.25	5.71	0.01	37.95	62.58	3.92	<0.01	46.72	72.45	3.92	<0.01
Ala	52.90	67.67	4.72	0.01	65.64	82.90	4.72	<0.01	48.31	66.82	3.35	<0.01	56.18	76.95	3.35	<0.01
Asp	30.52	50.53	6.78	0.02	47.61	70.45	6.78	0.01	30.73	47.09	5.42	0.01	44.92	64.96	5.42	<0.01
Cys	55.86	62.85	6.32	0.31	67.55	77.15	6.32	0.18	18.47	47.29	4.74	<0.01	30.09	56.60	4.74	<0.01
Glu	66.48	75.68	3.66	0.03	72.98	83.04	3.66	0.02	56.81	70.57	3.8	<0.01	60.86	75.48	3.80	<0.01
Gly	39.91	54.13	8.07	0.11	93.57	134.19	8.07	<0.01	17.01	24.82	9.8	0.44	59.88	90.78	9.80	0.01
Pro	64.03	76.10	3.97	0.01	70.76	83.11	3.97	0.01	57.18	70.55	3.98	0.01	61.10	75.22	3.98	0.01
Ser	46.20	56.87	6.67	0.14	63.34	81.41	6.67	0.02	32.68	50.6	5.92	0.01	46.70	69.89	5.92	<0.01
Tyr	48.90	58.55	7.54	0.23	58.26	70.19	7.54	0.15	39.47	62.5	4.13	<0.01	47.07	70.60	4.13	<0.01

AID: apparent ileal digestibility; SID: standard ileal digestibility; UF: unfermented; F: fermented; WL1: wine lees 1, strong-aroma grain; WL2: wine lees 2, soy-sauce-aroma grain; CP: crude protein; Arg: arginine; His: histidine; Ile: isoleucine; Leu: leucine; Lys: lysine; Met: methionine; Phe: phenylalanine; Thr: threonine; Trp: tryptophan; Val: valine; Ala: alanine; Asp: asparagine; Cys: cysteine; Glu: glutamic acid; Gly: glycine; Pro: proline; Ser: serine; Tyr: tyrosine.

**Table 8:** Apparent nutrient digestibility, digestible energy, and metabolizable energy of the two fresh distiller’s grains before and after fermentation in growing pigs.

Items	UF	F	SEM	*p*	UF	F	SEM	*p*
	**WL1**				**WL2**			
Dry Matter	86.92	87.59	0.94	0.49	78.91	93.31	0.52	<0.01
Crude Protein	86.54	86.95	1.11	0.72	72.22	91.75	0.84	<0.01
Ether Extract	85.72	85.67	2.35	0.99	79.52	92.93	1.51	<0.01
Crude Fiber	49.90	58.30	5.00	0.15	45.47	67.96	3.88	<0.01
Neutral Detergent Fiber	65.44	67.32	3.08	0.56	55.66	82.04	1.73	<0.01
Acid Detergent Fiber	48.54	52.37	5.39	0.50	71.76	75.94	1.99	0.06
Ash	63.79	66.22	3.24	0.47	60.11	85.38	1.12	<0.01
Digestible Energy	15.00	15.52	0.17	0.02	14.44	16.71	0.11	<0.01
Metabolizable Energy	14.90	15.41	0.17	0.01	14.27	16.59	0.11	<0.01

UF: unfermented; F: fermented; WL1: wine lees 1, strong-aroma grain; WL2: wine lees 2, soy-sauce-aroma grain.

## Data Availability

The data supporting the findings of this study are included within the article. Further inquiries can be directed to the corresponding author.

## Abbreviations

The following abbreviations are used in this manuscript:

AA	amino acid
ADF	acid detergent fiber
ADG	average daily gain
AID	apparent ileal digestibility
ANOVA	analysis of variance
BIAA	basal ileal endogenous amino acid
Ca	calcium
CF	crude fiber
CFU	colony-forming unit
CP	crude protein
CSD	corn-based basal diet
DE	digestible energy
DM	dry matter
EE	ether extract
F	fermented
GE	gross energy
FTIR	Fourier transform infrared spectrophotometer
KEGG	Kyoto encyclopedia of genes and genomes
LAB	lactic acid bacteria
LFF	liquid-state fermentation
ME	metabolizable energy
NDF	neutral detergent fiber
NFD	nitrogen-free diet
NRC	National Research Council
NSPs	non-starch polysaccharides
RSM	response surface methodology
SEM	standard error of the mean
SID	standardized ileal digestibility
P	phosphorus
PCoA	principal coordinates analysis
PTS	phosphotransferase system
UF	unfermented
WL	wine lees
WLD	wine lees diet
